# Efficacy and safety of using antibiotics to prevent post-operative complications in oral implant treatment: evidence-based review

**DOI:** 10.1038/s41405-023-00174-4

**Published:** 2023-10-31

**Authors:** Javed Ikram, Rawand Shado, Ines Novo Pereira, David Madruga, Haidar Hassan

**Affiliations:** 1 Rey Juan Carlos University, Av. de Atenas, S/N, 28922 Alcorcón, Madrid, Spain; 2 Barts & The London School of Medicine & Dentistry, Queen Mary University, Institute of Dentistry, Royal London Dental Hospital, Turner Street, London, E1 2AD UK; 3 University of Porto, Faculty of Dental Medicine, R. Dr. Manuel Pereira da Silva, 4200-393 Porto, Portugal; 4 Barts & The London School of Medicine & Dentistry, Queen Mary University, Centre for Cutaneous Research, Blizard Institute of Cell and Molecular Science, 4 Newark Street, Whitechapel, London, E1 2AT UK

**Keywords:** Dentistry, Dental implants

## Abstract

**Aims:**

To identify and critically appraise available evidence on the efficacy and safety of antibiotics in preventing complications following oral implant placement treatment.

**Methods:**

An electronic search was performed using PubMed, Ovid MEDLINE and Cochrane Library databases up to July/21 for the purpose of answering the research question: In[healthy adults treated with dental implants]the use of[different antibiotics before or immediately after treatment]in comparison to[treatment without antibiotics]is safe and effective in terms of[infection, pain, swelling, wound dehiscence, soft tissue healing, early/late implant failure]? Following the Best Evidence Topic methodology, the included studies were categorised based on the Oxford Centre for Evidence-Based Medicine (OCEBM) ratings. The critical appraisal skills programme CASP checklist was used for the methodological analysis. The risk of bias assessment was performed according to the Cochrane Methodology for Systematic Reviews of Interventions.

**Results:**

26 of the 245 initially identified articles met our inclusion criteria for analysis after applying rigorous filters. The included human studies demonstrated significant methodological heterogeneity, precluding meta-analysis. These studies spanned evidence levels II to IV, as per OCEBM 2011 classifications, with the United States contributing the most studies (19.2%, *n* = 5), all at level III. The United Kingdom and Spain followed with three studies each (11.5% each), two from the UK and one from Spain classified at level II. Most studies had less than 1 year of follow-up (21%). Our analysis included 26 studies, with 38 antibiotic patient groups totalling 7459 patients. Amoxicillin was the predominant antibiotic, with various dosage regimens. Complications were observed in studies across different amoxicillin regimens at a cumulative incidence of 5%.

**Conclusion:**

The evidence on antibiotics to prevent implant failure presents uncertain and heterogeneous findings. High-risk bias and underpowered studies were prevalent. Future research should prioritise multicentre, double-blinded RCTs with larger samples and longer follow-ups. Structured methodologies, antibiotic stewardship, and adherence to guidelines are needed. Amoxicillin (2 g) was commonly prescribed, but guidelines recommend 3 g, which results in relatively low complications yet there is limited evidence to support it. Clindamycin was favoured for penicillin allergies, but caution is advised due to potential implant failure risk. Consistent use of antiseptic mouthwash was observed. Future research should explore alternatives to antibiotics and antibiotic stewardship. Establishing a well-funded research consortium could yield conclusive results for clinical practice.

## Introduction

There is growing demand for dental implant treatments worldwide, with global figures of approximately $4.6 billion for 2022 and estimated to grow by 9.8% annually [1]. In Europe, the annual market is worth approximately $1.5 Billion [2]. It is thought that the increase in dental implant placements has led to an upsurge in antibiotic use [3]. Among the nations of Italy, the Netherlands, Spain, Sweden, and the United Kingdom, predominant antibiotic treatment regimens were observed [4]. The antibiotic regimen most frequently prescribed in Italy was amoxicillin, while in the Netherlands, the combination of amoxicillin and clavulanic acid prevailed [4]. Conversely, in Spain, no discernible antibiotic regimen exhibited predominance [4]. In Sweden, phenoxymethyl-penicillin held prominence, whereas in the United Kingdom, diverse alternative antibiotic regimens were notably favoured [4]. This wide variation in prescription rate for antibiotics reflects a global problem. Reportedly, dentists account for 10% of prescribers among healthcare professionals, with statistics varying across countries [5].

The World Health Organization (WHO) has issued a cautionary advisory, indicating that the excessive and improper prescription of antibiotics is leading a surge in antibiotic resistance. This phenomenon results in prolonged hospitalisation durations, elevated healthcare expenditures, and increased mortality rates [6]. Indeed, recent reports highlighted that in Europe, 25000 people die every year from antibiotic-resistant infections [7]. This could rise to 10 million a year by 2050, making it the number-one cause of death globally [7].

Recently, it was reported that the British National Health Service (NHS) spends around £30 billion per year to treat infections and infectious diseases [8]. This growing global crisis is partly due to prescribing practices, with half of all antibiotics used in human health care largely considered inappropriate [9]. Thus, several studies have focused on the dilemma of antibiotic resistance and whether prescribing antibiotics was effective for the intended use [10–12].

In the realm of dental implant treatments and antibiotic use, it is imperative to acknowledge the substantial body of existing knowledge. Several systematic reviews and research studies have explored the relationship between antibiotics and implant-related complications. These investigations have shed light on various aspects, including the types of antibiotics commonly prescribed, their dosages, and the timing of administration [13]. However, despite these valuable contributions, uncertainties and gaps in the current literature persist.

One notable uncertainty revolves around the effectiveness and necessity of antibiotics in preventing complications following dental implant placements. While some studies suggest a potential benefit in reducing postoperative infections [14], others raise questions about its benefits [15]. Moreover, there is a lack of consensus regarding the ideal antibiotic regimen, including the choice of antibiotic, dosage, and duration of treatment [13–15].

In the UK, the Faculty of General Dental Practice (FGDP) and the Faculty of Dental Surgery of the Royal College of Surgeons of England (FDS) guidance on antibiotic prophylaxis for dental implant placement appears to possess a degree of ambiguity [16]. The guidelines do not support the use of antibiotics for routine dental implant placement but recommend the use of Amoxicillin 3 g (or Clindamycin 600 mg), 1 hour before intervention, when providing intra-oral bone augmentation. It was inconclusive if the guidelines are intended to cover complex medical histories or vulnerable patients. Moreover, no guidance has been issued for multiple variables often seen in clinical practice, such as smoking, bisphosphonate medication, and immediate placement approaches. Hence, the clinicians have currently the difficult task of weighing up complex clinical scenarios while interpreting the limited guidance. This explains the importance of the present review to inform clinical practice of the best available scientific evidence and potentially promote a safer and effective clinical care.

There is little consensus among dental professionals regarding the ideal antibiotic type, combination regimen, dosage, timeframe (initiation and length) and route of administration [17–20]. Many surveys concluded that clinicians are strongly in favour of guidelines, protocols, and training that could help them optimise decision-making processes and to understand if and which antibiotics are required [21, 22]. This seems to be in line with the global emergency call to action to promote “antibiotic stewardship”, aiming at implementing adequate strategies for antimicrobials use through evidence-based approaches [23].

These uncertainties highlight the need for a comprehensive review that synthesises the existing evidence, clarifies conflicting findings, and provides guidance for clinicians facing these clinical dilemmas. This paper aims to address these gaps and uncertainties in the current literature by conducting an evidence-based review of the available evidence. By critically evaluating the methodology and findings of previous studies, we intend to provide a clear understanding of the clinical efficacy and safety of antibiotics in the context of dental implant treatments. Through this review, we seek to contribute valuable insights to the field and assist dental professionals in making informed decisions regarding antibiotic use in implant dentistry.

## Methods

**Fig. 1:**
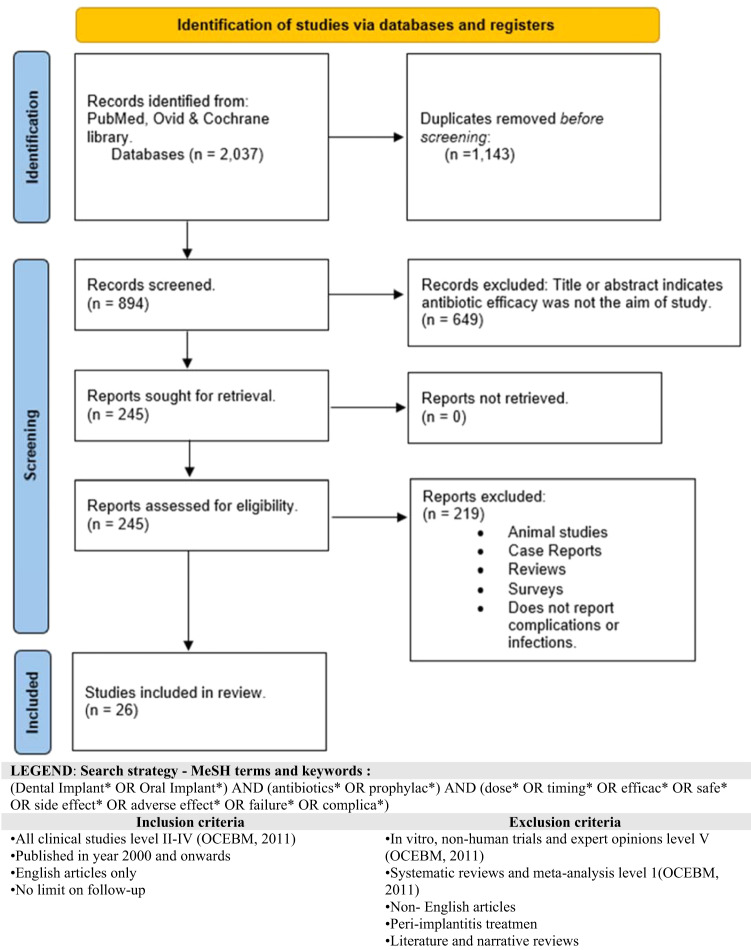
PRISMA flowchart. Shows The identification process for relevant article and their retrieval.

An evidence-based review was performed using advanced electronic search of PubMed, Ovid MEDLINE databases and Cochrane Library up to July 2021. Figure 1 illustrates the keywords and Booleans that have been used to screen for relevant papers. The outcome of this search was analysed and critically appraised using the Best Evidence Topic methodology (BestBETs) [24]. This tool was first pioneered by the Emergency Department at the Manchester Royal Infirmary, and it sets out to look at all the available evidence for a given clinical question. A more permissive methodology was adopted to broaden the inclusiveness of the eligibility criteria. The authors confirmed that incorporating studies which lack a control group, would offer diverse perspectives and comprehensively address the clinical query.

In conducting this review, it is important to clarify the methodology employed. While the term “Best BETs methodology” has been mentioned, we want to provide a transparent explanation of our approach. We are not exclusively adhering to the Best BETs methodology. However, our methodology incorporates elements of systematic review techniques to comprehensively assess the existing evidence.

Another important aspect of our approach was the decision to exclude level I evidence, which typically includes systematic reviews of randomized controlled trials (RCTs). This decision was grounded in our aim to assess primary data from the RCTs directly.

By excluding level I evidence, we aim to focus on a broader spectrum of research, including, RCTs, observational studies, cohort studies, case-control studies, and case-series. This approach allows us to consider a wider range of clinical scenarios and patient populations, providing a more holistic view of the subject matter. We critically appraised the quality of each included study and synthesised the evidence to offer practical insights into antibiotic use in dental implant treatments.

We sought to provide dental practitioners with a perspective on the clinical implications of antibiotics in implant dentistry, considering both the strengths and weaknesses of the available research.

No ethical approval was required as this is a review article with no original research data.

### Data selection

Two independent reviewers have systematically searched the databases and any disagreements on exclusion/inclusion criteria were resolved through consensus with a third and fourth reviewer. Figure 1 displays search parameters and results. The relevant studies were drawn into a BestBETs tables, while classified according to the type of study and assessed for their quality. The data extracted and appraised for each study, as well as the scoring, was confirmed through open discussion.

### Study quality and risk of bias assessment

The included studies were initially categorised in terms of level of evidence, following the OCEBM 2011 ratings [25]. The level of evidence hierarchy criteria consisted of: I-Systematic reviews of randomised control trials (RCTs); II-Individual RCTs with a narrow confidence interval; III-Cohort studies or low quality RCTs; IV-Case control studies, case series or poor-quality cohort studies ; V-Expert opinion. The I and V categories was excluded, given that none of the studies fitted our inclusion criteria.

The risk of bias assessment was performed for the included RCTs according to the Cochrane Methodology for Systematic Reviews of Interventions [26]. Each item was scored as low, unclear, or high risk of bias. The overall risk of bias for each RCT was determined based on the following: 1) low risk, if all domains were scored as “low” or only one as “unclear”; 2) high, if at least two domains were estimated “unclear”, or one domain was scored as “high”. Presentation and descriptive analysis were conducted using Microsoft Excel^®^ to tabulate and produce graphics.

### Data extraction and outcomes

Two reviewers independently extracted data on study design, study quality, country of publication, chronological distribution, and key outcomes most commonly reported in the different studies. Without restrictions, the type of implant and placement technique was reviewed in detail, as well as the follow-up period, the type of antibiotic and administration protocol and/or combination regimen. When reporting, data was extracted on clinical complications such as infection, pain, swelling, soft tissue healing, and early implant failure. Refer to Supplementary Table-1 for a brief description of the included studies conclusions, where findings were tabulated in chronological order with statistical significance or tendencies favouring antibiotics or not. In instances where a specific data point remains unreported within a given study, it was systematically documented as “NR”—an abbreviation denoting “Not Reported”—within the corresponding table.

### Data analysis and reporting

A meta-analysis was not possible to perform due to the heterogeneous nature of the outcomes in the included studies. Data was reported according to the PRISMA guideline [27]. The protocol of this review has not been registered. In every investigation, the antibiotic protocol and regimen were documented and subsequently visualised through implementation of a sunburst plot employing the Plotly library within the Python programming language. The rates of complications or infections were computed and subsequently depicted through a sunburst plot, employing a continuous colour scale to represent these rates in conjunction with their respective protocols.

## Results

The initial search yielded 245 articles, of which 26 were retrieved for analysis as per selection criteria shown in Fig. 1. Additional filters excluded non-English articles, reviews, comments, editorials, letters, consensus statements, animal trials, in-vitro studies, and irrelevant studies referring to peri-implantitis.

### Summary of findings

**Fig. 2:**
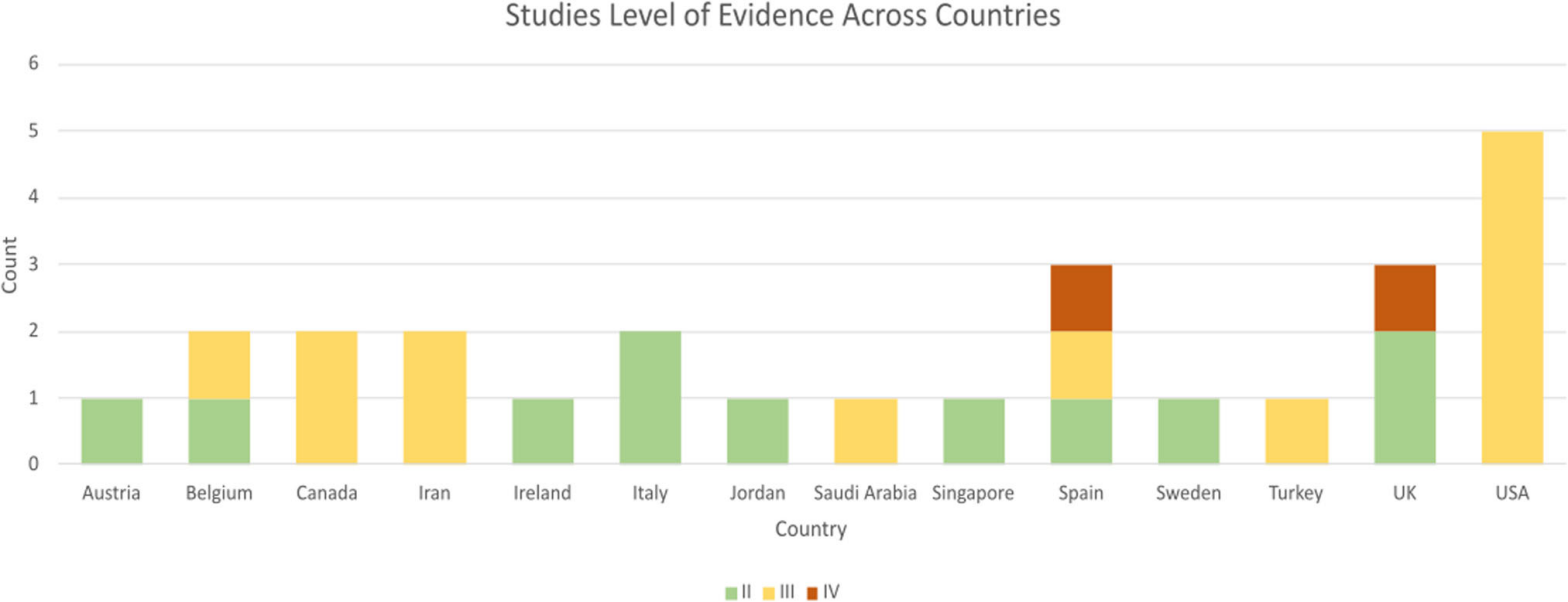
Studies Level of Evidence Across Countries. Bar chart representing the total number of studies identified in each contort with their corresponding level of evidence.

Statistical analysis of the 26 studies included in this evidence-based review revealed heterogeneous levels of evidence (II to IV), according to the OCEBM 2011 rankings. Data analysis considering the country-of-origin publication is displayed in Fig. 2 which showed that the United States displayed the most representation in terms of the number of studies conducted, accounting for 19.2% (5 out of 26). Notably, all studies originating from the United States were classified at level III of evidence. Meanwhile, the United Kingdom and Spain shared the second position with an equal count of three studies each, constituting 11.5% (3 out of 26) of the total. Furthermore, within this subset, two study from the United Kingdom and one from Spain were categorised at level II of evidence.

The follow-up period among the included studies was also evaluated to understand the time frame in which implant failures were considered. To this purpose, studies such as questionnaires were excluded. Thus, 26 studies were analysed and mostly presented follow-up periods of less than 1 year with some over 2 years or more.

**Fig. 3:**
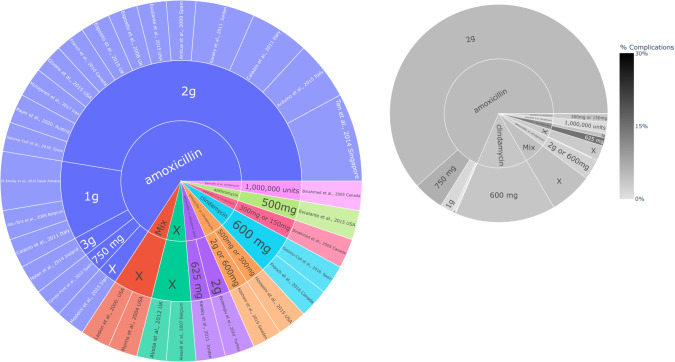
Antibiotic type and dose across the identified studies with complication rates. A sunburst plot showing the antibiotic used and onset dose in each study. Left plot is by study, right plot is by total participants. X = ‘missing data point’ LEGEND: Type of antibiotic → dose → Study (inner circle → outer circle).

**Fig. 4:**
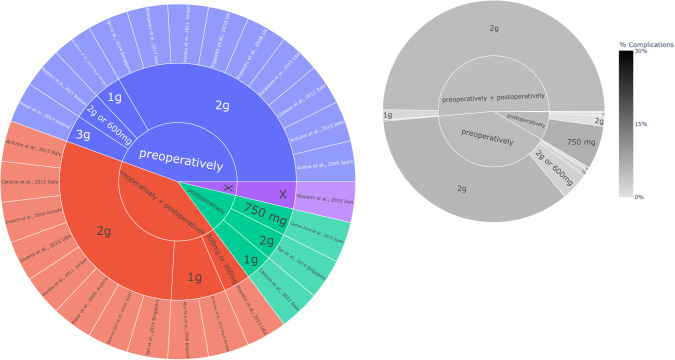
Amoxicillin protocol with complication rates across studies which used amoxicillin. A sunburst plot showing the amoxicillin protocol and dose in each study. Left plot is by study, right plot is by total participants. X = ‘missing data point’ LEGEND: Prescription time → dose → Study (inner circle → outer circle).

**Fig. 5:**
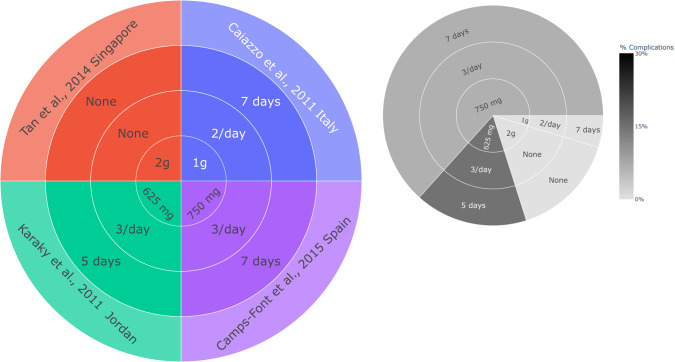
Amoxicillin Postoperative protocol with complication rates across studies prescribing amoxicillin postoperatively. A sunburst plot showing the amoxicillin postoperative protocol. Left plot is by study, right plot is by total participants. LEGEND: Postoperative antibiotic dose → frequency → antibiotic postoperative protocol duration → study (inner circle → outer circle).

The analysis included 26 studies with 38 antibiotic patient groups, comprising a total size of 7459 patients who were administered antibiotics. Amoxicillin stands out as the predominant antibiotic in terms of both the quantity of studies (69.23%, 18/26), antibiotic groups (65.79%, 25/38), and the total number of participants involved (65.82%, 5111/7459), (see Figs. 3 and 4). Most investigations incorporated amoxicillin in a combined preoperative and postoperative regimen, with some providing it solely in the preoperative phase, and a smaller subset exclusively in the postoperative phase (Fig. 5). The dosages of amoxicillin varied between 750 mg and 3 g, with 2 g emerging as the prevailing choice in relation to both the number of studies conducted and the cumulative participant count. The solitary study adhering strictly to the protocol outlined by the FGDP guideline exhibited an absence of complications, recording a rate of 0%. (Fig. 4)

The cumulative incidence of complications observed across studies providing postoperative, preoperative, and perioperative + postoperative amoxicillin regimens each stands at 5%.

### Risk of bias assessment

The risk of bias assessment for the included 13 RCTs since 2000 is illustrated in Table 1. The evaluation of each study consistently showed that the required data was either missing, unclear or in a format of difficult interpretation. Differing presentation layouts between journals made the task of identifying the relevant data more onerous.

**Table 1:** Risk of bias in included RCTs.

RCTs	Selection bias (Randomisation)	Performance bias (Blinding of participants and personnel)	Detection bias (Blinding of outcome assessment)	Attrition bias (Incomplete outcome data)	Reporting bias (Selective reporting)	Overall score
Payer, 2020	Low	Low	Low	Low	Low	Low
Kashani, 2019	Low	Unclear	Unclear	Low	Unclear	High
Escalante, 2015	Low	Low	Low	Low	Low	Low
Arduino, 2015	Low	Low	High	Low	Low	High
Hosseini, 2015	Low	Low	Low	Unclear	Low	Low
Nolan, 2014	Low	Low	Unclear	Unclear	Unclear	High
Tan, 2014	Low	Low	Unclear	Unclear	Unclear	High
Karaky, 2011	Low	High	Low	Low	Unclear	High
Caiazzo, 2011	Low	Unclear	Unclear	Unclear	Unclear	High
Esposito, 2010	Low	Low	Low	Low	Low	Low
Anitua, 2009	Low	Low	Low	Low	Low	Low
Esposito, 2008	Low	Low	Low	Low	Low	Low
Abu-Ta´a, 2008	Low	Low	Low	Low	Low	Low

The overall risk of bias: Low, if all domains were scored as “low” or only one as “unclear”; High, if at least two domains were estimated “unclear”, or one was scored as “high”.

## Discussion

This evidence-based review sought to highlight the current gaps surrounding the prescription on antibiotics for oral implant treatment. In general, there was considerable heterogeneity among the included studies, which revealed different baseline characteristics (e.g., risk factors), a variety of sample sizes and methodologies reported. Overall, 50% (13 out of 26) of the studies were RCTs characterised by a level II of evidence, with two exceptions being categorised as level III due to their limited sample size. The remaining studies were predominantly categorised at level III or below in terms of evidence hierarchy. Among the subset of RCTs, 53.8% (7 out of 13) were identified as having a low risk of bias, while the remaining were assessed to be at a high risk of bias. There was wide variation in antibiotic dosages, administration timings, and recommended length for the taking, with many other confounding variables that may have influenced conclusions (e.g., the level of asepsis). Overall, considering all these variables, the characteristics and risk of bias of the included studies, the results of a clinical added benefit of antibiotics in preventing postprocedural complications in oral implantology need to be interpreted with caution.

Amoxicillin was the number one prescribed antibiotic, often via oral administration and pre-operatively (82%). The dose ranged between 1-3 g, however, 2 g was the most commonly reported among the included studies, which is in parallel with the FGDP/FDS recommendations for a dose of 3 g. Clindamycin was the second most prescribed antibiotic, particularly in scenarios of reported allergy to penicillin. However, two studies observed an increased risk of implant failure when pre- and post-operative clindamycin were jointly prescribed [28, 29]. Further research is required to confirm the minimum inhibitory concentrations [30], the effect on osseointegration, or whether an alternative antibiotic (e.g., azithromycin) could be used as an alternative [31]. It also remains unclear if the risk of implant failure with a single pre-operative dose of antibiotic and a combined approach is comparable. Antiseptic mouthwashes were confirmed in all the studies, but there was lack of standardised protocols on timings, amount, length, time of rinsing, or even delivery mode, i.e., whether a gel or mouthwash formulation was favoured [32].

The use of post-operative antibiotics did not significantly reduce post-operative infections or implant failures. There was no agreement between studies on the best protocol for post-operative antibiotics. No evidence was found on the potential combination of antibiotics, and the reason why these would not be suitable remains to be elucidated. Safety and adverse drug reactions were generally under reported. Noteworthy that there was also high degree of heterogeneity in procedure technique, such as flapless, open flap with and without bone graft, sinus augmentation, immediate or delayed placement, loaded versus not loaded, involving infected and non-infected sockets. There was also great variation regarding the location for the implant placement, including the maxilla, mandible, anterior/posterior arcade, near and distant from the remaining teeth; These are all independent factors with the potential to impact dental implant success rates, regardless of the antibiotic use. The occurrence rate of complications, as determined from studies providing postoperative, preoperative, and perioperative + postoperative amoxicillin regimens, consistently stands at 5%. Nevertheless, in order to conclusively affirm discrepancies in outcomes associated with each respective protocol, the conduct of a meta-analysis becomes imperative, facilitating a thorough and comprehensive evaluation.

The singular study that closely adhered to the FGDP guideline protocol was conducted in Ireland, wherein a preoperative administration of 3 g of amoxicillin was implemented, yielding a complication rate of 0%. However, it’s noteworthy that the sample size for this study was relatively modest, consisting of 27 participants. This figure accounts for a mere 0.053% of the combined sample size across all studies (27/5111). To definitively validate the viability of administering 3 g of amoxicillin preoperatively, further randomized controlled trials with larger sample sizes within the antibiotic subgroup are essential.

There were further limitations among the included studies preventing more meaningful conclusions. For example, exclusion criteria varied, but typically included medically compromised patients, cases requiring prophylactic antibiotics, alcoholics, presence of untreated dental disease, pregnancy, radiation treatment, diabetes, lack of minimum bone grafting required, immunodeficiency, intravenous bisphosphonates, smokers, and penicillin allergy. These could all have had a dramatic effect on implant success rates and primary endpoints. Moreover, it is yet unknown if the age and gender variables can also contribute to post-operative complications, including implant failure. Conversely, untreated periodontal disease has long been well-recognised as a risk factor for implant failure [33]. In addition, one study presented important clinical content by measuring and quantifying the experience of implant surgeons [34]. It was concluded that surgeons with clinical experience of 50 implants or more had lower implant failure rate when compared to surgeons with less than 50 implants treatment record (2.9% and 7.3%, respectively) [34]. The same study observed that the likelihood of implant failure increased with prolonged implant surgery duration. Similarly, the number of implants placed in a single intervention was directly proportional to the implant failure rate. Interestingly, the confounding variable consistently used in all RCT studies was the chlorhexidine gluconate or digluconate, as a mouthwash (0.12% or 0.2%) or dental gel (1%), while iodine was less reported. The length of time for their use ranged from one to eight weeks, and up to three times a day, consisting of either rinsing pre-operatory, post-operatory, and/or both. Despite the lack of widely applicable standardised protocols for the use of these antiseptics [32], there is compelling evidence to demonstrate that they have been successfully reducing bacteraemia in implant placements when used pre-operatively. [35]While not the primary objective of this investigation, an intriguing observation emerged. The use of pre-operative 0.12% chlorhexidine mouthwash exhibited an associated reduction of up to 10% in implant failure rates [36].

Finally, adverse drug reactions were under reported and mostly not considered as primary outcome measures. Only four studies have specifically measured adverse drug reaction clinical parameter [37–40].

### Limitations

Several limitations are evident within the scope of this review. The inclusion criterion restricted the selection to studies available exclusively in the English language, potentially introducing a language bias. Furthermore, the temporal range of considered articles, spanning from 2001 to 2021, might not encompass all relevant developments in the field. The inherent heterogeneity among the chosen papers, possibly stemming from variations in methodologies, populations, or interventions, introduces a source of potential inconsistency in the synthesis of results. Additionally, an acknowledged constraint is the presence of a certain degree of bias risk within the selected papers, which could influence the overall validity and reliability of the synthesized findings.

## Conclusions and future directions

The current body of evidence regarding antibiotics use for preventing implant failure remains inconclusive and exhibits inherent weaknesses. The available evidence supports the administration of preoperative antibiotics as opposed to postoperative administration, as well as, indicating a lower incidence of complications associated with antibiotic administration compared to non-antibiotic regimens. Nevertheless, we must note the strength of the available evidence does not merit clear extrapolations.

To address this, there is a pressing need for structured research methodologies and a comprehensive approach to antibiotic stewardship. This entails a reduction in unjustified antibiotic prescriptions and the rigorous implementation of aseptic protocols in clinical practice. While Clindamycin is favoured in cases of penicillin allergies, caution is warranted due to the potential risk of implant failure associated with its combined pre- and post-operative use.

### Supplementary information

Supplementary Information

## Data Availability

The data that support the findings of this study are available from the corresponding author upon reasonable request.
