# Supplementary material for: Efficacy and safety of using antibiotics to prevent post-operative complications in oral implant treatment: evidence-based review
Source: BDJ Open. 2023 Oct 31;9:47. doi: 10.1038/s41405-023-00174-4 (PMC10618562; doi:10.1038/s41405-023-00174-4)
Supplement: Supplementary file 1 — Supplementary Information [file 41405_2023_174_MOESM1_ESM.pdf]

SI Table 1. BestBets table representing summary characteristics and finding for each study.

Author, Country, Year	Study Type, Level of Evidence	Population	AB protocol	Evaluation (Follow up)	Type of implant cases	Outcomes measures	Key results	Study Strength	Study Weakness
Bedeloglu et al., 2020 Turkey	Cohort Study III	Total: 75<br>male: 30<br>female: 45<br>implants: 123<br>Age: 46.17 ± 14.79	Pt: 50<br>Delivery: tablet<br>Type: amoxicillin + clavulanic acid<br>Onset dose: 2g<br>Onset: preoperatively<br>Continuation dose: 1g<br>Frequency: 2/day<br>Duration: 5 days	12,7,14 days and 12th week	Simple cases	Is perioperative antibiotic necessary in straightforward implant placement procedures?<br>Pain, swelling, bleeding, cyanosis, flap dehiscence, suppuration, IFR	1.AB use has no effect on IFR<br>2. No SS regarding pain, swelling, bleeding, cyanosis, flap dehiscence, suppuration		1. Low sample size<br>2. Lack of randomisation
Payer et al., 2020. Austria	RCT II	Total: 236<br>male: 125<br>female: 111<br>implants: 236<br>Age: 46	Pt: 117<br>Delivery: oral<br>Type: amoxicillin<br>Onset dose: 2g<br>Onset: preoperatively + postoperatively<br>Continuation dose: 500mg<br>Frequency: 3/day<br>Duration: 3 days	7-21 days with variation	Single and multiple	A prospective observational study on perioperative use of antibacterial agents in implant surgery<br>Plaque levels	1. Use of antiseptic mouthwashes and gels could be used as an alternative to antibiotics to facilitate wound healing.<br>2. 1. AB's 54% of patients pre-op upto 67% had antiseptics given post implant upto 94.7% given AB's and m/wash upto 86.5% gel upto 100%<br>3. Practice guidelines covering asepsis provision would be useful	1. Selection bias minimised.<br>2. Ethical approval gained in 3 countries. 1 country it was waived.	1. Funding by Pierre Fabre Medicament. 2. Authors have received consulting fees from above<br>3. No IFR & no distinguishing AB type or dosage.

Kashani et al., 2019 Sweden	RCT II	Total: 447<br>male: 109<br>female: 114<br>implants: 963<br>Age: 56 ± 17.7	Pt: 223<br>Delivery: tablet<br>Type: amoxicillin or clindamycin<br>Onset dose: 2g or 600mg<br>Onset: preoperatively<br>Continuation dose: None<br>Frequency: None<br>Duration: None	4 month follow up	Single and 2 stage placements with and without bone grafting	Influence of a single preoperative dose of antibiotics on the early implant failure rate. A randomised clinical trial<br>Implant failure rate	1. SS EIFR assessing implant numbers -AB group 2.2% v no AB's 7.5% P = 0.0011<br>2. Patient numbers AB group 4.9%% v no AB's 12.9% P = 0.0045<br>3. 3-4 times failure risk(3.38 on odds ratio at 95% CI)	1. Approved ethics committee Gothenburg University<br>2. Bias risk - reduced pt and surgeon allocation limited to 1 surgeon<br>3. Surgeons were experienced<br>4. Exclusion and Inclusion criteria defined.<br>5. Confidence level 95% = power 80%	1. Funding not declared
Salomo-Coll et al., 2018. Spain	RCT II	Total: 1210<br>male: 665<br>female: 545<br>implants: 2747<br>Age: NR	Pt: 1133<br>Delivery: tablet<br>Type: amoxicillin<br>Onset dose: 2g<br>Onset: preoperatively + postoperatively<br>Continuation dose: 750 mg<br>Frequency: 3/day<br>Duration: 7 days	4 month follow up	Single and 2 stage placements with and without bone grafting	Influence of a single preoperative dose of antibiotics on the early implant failure rate. A randomised clinical trial<br>Implant failure rate	1. SS EIFR assessing implant numbers -AB group 2.2% v no AB's 7.5% P = 0.0011<br>2. Patient numbers AB group 4.9%% v no AB's 12.9% P = 0.0045<br>3. 3-4 times failure risk(3.38 on odds ratio at 95% CI)	1. Approved ethics committee Gothenburg University<br>2. Bias risk - reduced pt and surgeon allocation limited to 1 surgeon<br>3. Surgeons were experienced<br>4. Exclusion and Inclusion criteria defined.<br>5. Confidence level 95% = power 80%	1. Funding not declared

			Pt: 77<br>Delivery: tablet<br>Type: clindamycin<br>Onset dose: 600 mg<br>Onset: preoperatively + postoperatively<br>Continuation dose: 300 mg<br>Frequency: 4/day<br>Duration: 7 days						

Mohajerani et al., 2017<br>Iran	Cohort Study<br>III	Total: 1093<br>male: 388<br>female: 705<br>implants: 1093<br>Age: 20-60	Pt: 1037<br>Delivery: tablet<br>Type: amoxicillin<br>Onset dose: 2g<br>Onset: preoperatively<br>Continuation dose: None<br>Frequency: None<br>Duration: None	N/A	No bone grafting, smoking or immunocompromised.<br>2 groups<br>1. Pt's with a failed implant before loading<br>2. Pt's without a failed implant before loading	The Risk Factors in Early Failure of Dental Implants: a Retrospective Study<br>Age, gender, implant type, implant surface, implant length, bone type, type of surgery (one- or two-stage) and immediate (fresh socket) or delayed placement of implant were the variables to be assessed in this study.	1. Implant surface, fresh socket placement, prophylactic use of AB's, and bone density were all significant risk ($p< 0.05$)<br>2. IFR was 6.68%<br>3. No gender, age group or implant length were significant.<br>4. Bone density IFR= D1- 5% D2- 2.2% D3-13.6% D4-19.2%<br>5. AP- 4.6% v no AP- 44.6%	1. Ethics approval from Shahid Behesti University of medical sciences<br>2.Exclusion criteria good<br>3. Inclusion pt's- 1.9.08- 31.10.2015- 7 years<br>4. Funding not stated<br>5. Smoking was excluded	1. Heterogeneity of bone density to implant length will have a bearing on success<br>2. Experience of surgeons not stated there is a heterogeneity of clinical backgrounds<br>3. Funding not stated

French et al., 2016 Canada	RCT II	Total: NR<br>male: NR<br>female: NR<br>implants: 5576<br>Age: 60 (20-89)	Pt: NR<br>Delivery: tablet<br>Type: amoxicillin<br>Onset dose: 2g<br>Onset: preoperatively + postoperatively<br>Continuation dose: 500mg<br>Frequency: 3/day<br>Duration: 7 days	1. 1 Year follow- up for implants	All types including bone grafting	Self reported penicillin allergy and relation to IFR and infections. Allergy to penicillin and IF both EIF and LIF	1. Confounders inc IFR- periodontal disease, smoking 15 a day, history of diabetes, Immediate implantation.<br>2. Allergy to penicillin = IFR 3.1 times.<br>3. Immediate IFR was x10 times in penicillin Allergic groups<br>4. Infection rate was x6 higher with penicillin allergic group<br>5.Clindamycin = -ve effect on osteoblasts in vitro.<br>6. Allergy testing?<br>7. Higher IFR in 7's<br>6. Clindamycin not as effective as penicillin.	1. Graft, , sinus, immediate had post op AB's<br>2. 1 experienced periodontist<br>3. Ethics approval University of British Columbia<br>4. Bias -single clinician and retrospective study	1. No post op dose with Penicillin allergy patients<br>2.Wide heterogeneity of clinical cases including systemic disease, smoking.<br>3. Funding not disclosed but work set in private practice not university
			Pt: NR<br>Delivery: tablet<br>Type: clindamycin<br>Onset dose: 600 mg<br>Onset: preoperatively<br>Continuation dose: None<br>Frequency: None<br>Duration: None						

Camps-Font et al., 2015 Spain	Case-control analysis IV	Total: 337<br>male: 0<br>female: 0<br>implants: 1273<br>Age: 0	Pt: 337<br>Delivery: tablet<br>Type: amoxicillin<br>Onset dose: 750mg<br>Onset: postoperatively<br>Continuation dose: 750mg<br>Frequency: 3/day<br>Duration: 7 days	42.9 months - 3.58 years	No GBR	Postoperative Infections After Dental Implant Placement: Prevalence, Clinical Features, and Treatment.<br>The prevalence and describe the clinical features and treatment of patients with early infections after implant placement.	1. 4-10% pt's develop post op infections and of these 66% the infected implant fail.<br>2. Late onset postoperative infections (1 month after placement) are difficult to treat, as 66% of implants with this complication failed.<br>3. Multiple factors were checked such as clinician experience, smooth or rough surface, lack of soft tissue coverage<br>4. There was a AB resistance to treatment when there was an infection<br>5. Paper published in 2015 time assessed cases from 2009 onwards so a 6 year period.	2. Inclusion criteria: consecutive patients 2.2009-10.2012 Smokers, periodontitis, diabetes pt's included. ASA I,II,III<br>4. Ethical approval-University of Barcelona<br>6. Exclusion ASAmore than III<br>7. Funding-Dental and Maxillofacial Pathology and Therapeutic" research group of the IDIBELL Institute.	1. Post -op AB's given<br>5. Experience - 3rd year MSc students in Oral surgery and Implantology<br>8. Bias- this is a retrospective study<br>2. Original 452 pt's 115 excluded due to incomplete records, grafting techniques,6 unconventional loading, 11 previous failure location.

Escalante et al., 2015 USA	RCT III	Total: 13<br>male: 7<br>female: 6<br>implants: 13<br>Age:	Pt: 7<br>Delivery: tablet<br>Type: amoxicillin<br>Onset dose: 2g<br>Onset: preoperatively<br>Continuation dose: None<br>Frequency: None<br>Duration: None	20 days post op.<br>1 hour pre-op, 6, 13, 20 days	Single stage dental Implant placements	Comparison of Azithromycin and Amoxicillin Before Dental Implant Placement: An Exploratory Study of Bioavailability and Resolution of Postoperative Inflammation.<br>Studies suggest that a single prophylactic dose of Am reduces early implant complications, but it is unclear whether other AB's are also effective. This study compared the local antimicrobial and anti-inflammatory effects resulting from a single dose of Azithromycin or Am before surgical placement of one-stage dental implants.	1. Azithromycin was available longer than Am at surgical site.<br>2. Azithromycin had less cytokines and chemokines in GCF and PICF	3. Inclusion criteria: Non smoker >21 year old, 2 adjacent teeth, no systemic disease, allergy not pregnant not taken drugs 1 month prior to study.<br>5. Funding - 3 grants 2 from Ohio State University and NIDCR Bethesda<br>6. Exclusion everyone outside the inclusive criteria	1. Small sample size to get a power of 80%<br>2. No control due to ethical standards.<br>4. Experience not stated<br>7. Bias- clinicians sampling not stated.<br>8.Post -op chlorhexidine 0.2% given and no use of NSAID's

			Pt: 6<br>Delivery: tablet<br>Type: azithromycin<br>Onset dose: 500mg<br>Onset: preoperatively<br>Continuation dose: None<br>Frequency: None<br>Duration: None						
Arduino et al., 2015 Italy	RCT II	Total: 360<br>male: 171<br>female: 189<br>implants: 567<br>Age: 50.45 ± 14.13	Pt: 166<br>Delivery: tablet<br>Type: amoxicillin<br>Onset dose: 2g<br>Onset: preoperatively<br>Continuation dose: None<br>Frequency: None<br>Duration: None<br><br>Pt: 177<br>Delivery: tablet<br>Type: amoxicillin<br>Onset dose: 2g<br>Onset: preoperatively + postoperatively<br>Continuation dose: 1g<br>Frequency: 2/day<br>Duration: 2 days	6 months	Simple cases no bone grafting	Single preoperative dose of prophylactic amoxicillin versus a 2-day postoperative course in dental implant surgery: A two-centre randomised controlled trial. Prosthetic and IF, adverse effects and post op complications	1. No SS differences were observed between 2 g of preop Am and an additional 2-day postop course.	3. Experience 2 experienced dentists in private clinics<br>5. Exclusion criteria- patients having implanted biomaterials in the body (hip or knee prostheses etc); with a clinically significant medical history those who received radiotherapy to the head and neck area; patients in need of bone augmentation proced- ures concomitant with implant placement; allergic to penicillin; already under on AB for any other reasons; patients treated or under treatment with IV bisphosphonates; pregnant or lactating females. 6. Inclusion - Consecutively healthy pt's	1. Control group AB's given were lower than normal and not for a 5 day course. 2. Loss of data for 21 patients, 6% of total. 4. Adverse effects in post op AB group. 7. Sample size was small<br>8. Funding- pt's from private practice.<br>9. Only apply to non grafting cases.

Hosseini et al., 2015 USA	RCT III	Total: 20 male: 9 female: 11 implants: 20 Age: 33-72	Pt: 10<br>Delivery: tablet<br>Type: amoxicillin or clindamycin<br>Onset dose: 500mg or 300mg<br>Onset: preoperatively + postoperatively<br>Continuation dose: 500mg or 300mg<br>Frequency: 4/day<br>Duration: 7/days	1 & 4 w post surgery. 6 month post implant placement	Immediate placement with teeth which had periapical lesions	Effects of AB's on Bone and Soft-Tissue Healing Following Immediate Single-Tooth Implant Placement Into Sites With Apical Pathology.	1. AB's had no effect on immediate implant outcomes on bone and soft tissue,	1. Very low sample size of 20, 10 and 10.<br>4. Bias- double blind study<br>5. Ethical approval- University of North Carolina<br>6. Inclusion - ASA I & II<br>7. Exclusion ASA III or above or taking AB's steroids, immunosuppressive drugs. 8. Funding Not stated.	1. Very low sample size of 20.<br>3. Did not measure IFR just bone and soft tissue healing. The 2 cases of IF was due to overloading and provisional restoration high on the occlusion.<br>8. Funding not stated.

Givens et al., 2015 USA	Cohort Study III	Total: 13<br>male: 7<br>female: 6<br>implants: 13<br>Age: NR	Pt: 5<br>Delivery: tablet<br>Type: amoxicillin<br>Onset dose: 2g<br>Onset: preoperatively + postoperatively<br>Continuation dose: 500mg<br>Frequency: 3/day<br>Duration: 7 days	1, 4 weeks then 6,12 months post implant placement	Immediate placement and provisionalisation into areas of peri-apical infection<br>Implant needed to have 50Ncm torque at placement for loading	Immediate Placement and Provisionalization of Implants Into Sites With Periradicular Infection With and Without AB's: An Exploratory Study	1. AP administration in healthy subjects does not appear to have a positive effect on the survival rates of implants placed into such sites, although further large-scale trials are needed to validate these findings. Need to take these results with caution as small sample and uneven groups	4. Inclusion ASA I & II smokers.<br>5. Exclusion ASA III & IV, on AB's, purulent discharge, use smokeless tobacco.<br>6. Ethical approval- North Carolina Chapel Hill university	1. Power is very weak sample size small<br>2. Both groups given Chlorhexidine 0.12% pre-op and during surgery socket rinse and post rinse<br>3. Bias and Funding supported by Zimmer Dental and part by American Academy of Implant Dentistry Research Grant
Moslemi et al., 2015 Iran	Cohort Study III	Total: 20<br>male: 6<br>female: 12<br>implants: 20<br>Age: 46.45 ± 13.03	Pt: 10<br>Delivery: tablet<br>Type: amoxicillin<br>Onset dose: NR<br>Onset: NR<br>Continuation dose: NR<br>Frequency: NR<br>Duration: NR	1,3,6 months after Implant placement	Non submerged<br>Age 20-60y<br>No bone graft	The Efficacy of Long-Term Post-Operative Antibiotic Therapy Versus Placebo on Dental Implants<br>Objectives: post-op morbidity and IF	1. No SS between groups on swelling and post op pain. 2. IF occurred in 2 patients placebo group =8.9% higher but authors stated this was not SS result.	3. Inclusion pt's 20=60 partially a edentulous, implant non-submerged<br>4. Exclusion criteria bone grafting submerged implant placement patients with poor OH, smokers, history of perio any systemic condition use of antibiotics. 4. Funding - Tehran University.<br>5. Bias- triple blind trial	6. Ethical approval - not stated.<br>7. Post op Chlorhexidine 0.2% used bd for 1 week.

El-Kholey et al., 2014 Saudi Abrabia	Cohort Study III	Total: 80<br>male: 30<br>female: 50<br>implants: 90<br>Age: 31.1 ± 7.3	Pt: 40<br>Delivery: tablet<br>Type: amoxicillin<br>Onset dose: 1g<br>Onset: preoperatively<br>Continuation dose: None<br>Frequency: None<br>Duration: None	3 days, 1 week, 12 weeks	Simple 2 stage without bone grafting	Efficacy of two antibiotic regimens in the reduction of EIF: a pilot study. A preop single-dose regimen and a 3-day AB course were compared. 80 subjects needing dental implant placement were randomly allocated to one of the two AP regimen groups	1. No SS between the 2 groups in terms of IFR	1. Low sample size between the 2 groups 2. Chlorhexidine 0.12% and used post op 3. Ethical approval - Ibn Sina College. 4. Funding - none 5.Inclusion- patients attending 9.2010-12.2012 6. Exclusion- Taken or required AB's, pregnant, bone graft, <18 , have systemic disease. 7. Experience- All implant procedures carried out by author 8. Bias- Not stated if author was blinded. 9. Adverse effects- none noted.	
			Pt: 40<br>Delivery: tablet<br>Type: amoxicillin<br>Onset dose: 1g<br>Onset: preoperatively + postoperatively<br>Continuation dose: 500mg<br>Frequency: 3/day<br>Duration: 3 days						

Nolan et al., 2014 Ireland	RCT II	Total: 55<br>male: 19<br>female: 36<br>implants: 82<br>Age: 56% <40, 31% 40-60, 13% >60	Pt: 27<br>Delivery: tablet<br>Type: amoxicillin<br>Onset dose: 3g<br>Onset: preoperatively<br>Continuation dose: None<br>Frequency: None<br>Duration: None	2 and 7 days osseointegration check at 3-4 months	Simple<br>no bone graft<br>No immediate placements	The influence of AP administration on post-op morbidity in dental implant surgery. A prospective double blind RCT	1. Post op pain is SS reduced in AB group. At 2 and 7 days<br>2. Higher IFR (100% v 82%) in placebo group.<br>3. Bruising was SS higher in placebo group 4. Longer the surgery the higher the failure rate 5. More implants placed higher the failure rate 6. Surgeons with >50 implants v <50 implants IFR 2.9%v 7.3%<br>7. High level pain indicating interference with daily activities indicator of failing osseointegration.	3. Inclusion- Presence of a partial edentulous/edentulous alveolar ridge.<br>teeth non-restorable with the intention of immediate implant placement.<br>Periodontally healthy remaining dentition.<br>non-infected surgical site.enough bone and soft tissue for the implant to be placed without additional augmentation procedures. Smoking included<br>4. Exclusion- All medical systemic conditions, allergy to penicillin, bone graft and non compliance excluded.	1. Low sample size<br>2. Chlorhexidine 0.12% and used pre-op and post op for 1 week 4-5 times a day! 3. 4 separate implant systems used.<br>6. Adverse effects not noted<br>7.Experience - post grad perio students<br>8. No sample size calculation done<br>9. Funding not stated<br>10. Ethical approval- Not stated.

Tan et al., 2014 Singapore	RCT II	Total: 329<br>male: 182<br>female: 147<br>implants: NR<br>Age: NR	Pt: 81<br>Delivery: tablet<br>Type: amoxicillin<br>Onset dose: 2g<br>Onset: preoperatively<br>Continuation dose: None<br>Frequency: None<br>Duration: None	Post implant placement 1,2,4,8weeks	number of implants Not stated. 1 implant system simple no bone graft	Effect of systemic AB's on clinical and PROM's of implant therapy - a multi centre RCT<br>Primary outcome variable: Patient-reported outcomes: VAS score on pain, swelling, bruising and bleeding.<br>Secondary outcome variables: Clinical recordings of flap closure, pain, swelling, suppuration, and implant stability by calibrated examiners.	1. SS poor flap closure at 4 weeks on Group 4 v compared to other groups 5% v 0%<br>2. All AB regimens do not improve PROMS or post surgical complications.-bruising, swelling, bleeding, or pain	2. 1 implant lost in Group 4 non in AB group<br>3. Chlorhexidine used pre-op not stated post op.<br>4. Ethical approval - gained from 7 institutes<br>5. Funding-<br>6. Inclusion- ASA I-II >19, non smokers, light smokers <20 a day, no allergies to Am or penicillin	1. Evaluation only 8 weeks looking at PROM's not IFR<br>2. Only single blind -surgeons and examiners but pt's were not.
			Pt: 82<br>Delivery: tablet<br>Type: amoxicillin<br>Onset dose: 2g<br>Onset: postoperatively<br>Continuation dose: None<br>Frequency: None<br>Duration: None						
			Pt: 86<br>Delivery: tablet<br>Type: amoxicillin<br>Onset dose: 2g<br>Onset: preoperatively + postoperatively<br>Continuation dose: 500mg<br>Frequency: 3/day<br>Duration: 2 days						

Alissa et al., 2012 UK	Case-control analysis IV	Total: 83<br>male: 32<br>female: 51<br>implants: 290<br>Age: 51 ± 18.7	Pt: 17<br>Delivery: NR<br>Type: NR<br>Onset dose: NR<br>Onset: NR<br>Continuation dose: None<br>Frequency: None<br>Duration: None	1-2 years	All types including iliac crest graft	Influence of prognostic risk indicators on osseointegrated dental IF: a matched case-control analysis.<br>Outcome looked at IFR in different categories.	1. SS smokers compared to non smokers IFR was higher<br>2. Alcohol >5 units / day SS higher IFR 3. Post op AB's SS reduced IFR<br>4. SS Higher diameter reduced IFR<br>5. Complications were SS reduced with post op AB's.<br>6. Implant loss before loading =41%(early failure) late failure = 59% after loading	1. Inclusion- Pt's at MDH 2.2000-12.2006 that they had at least 1 failed implant<br>2. Exclusion- Inadequate or unavailable records<br>4. Adverse effects- N/A as retrospective<br>6. Power analysis was 80%<br>7. Bias reduced by matching pt's in control group to study group.	3. Funding- Not stated<br>5. Experience- Not stated
Karaky et al., 2011 Jordan	RCT II	Total: 240<br>male: NR<br>female: NR<br>implants: 856<br>Age:NR	Pt: 73<br>Delivery: tablet<br>Type: amoxicillin<br>Onset dose: 2g<br>Onset: preoperatively<br>Continuation dose: None<br>Frequency: None<br>Duration: None	1 week 1 month prior to prosthesis fit 3-4 months later	All types and single to multiple sinus augmentation and standard augmentation.	AP and early dental IF: a quasi-random controlled clinical trial.<br>Comparing 3 regimens Group A (2 g Am single preoperative dose), Group B (single preoperative 2 g Am followed by 500 mg three times daily for 5 days) and Group C (postoperative Am with Clavulanic acid 625 mg three times daily for 5 days).	1. No SS between group A, B, C<br>2. Pre and post op AB's may have no significance compared to just pre-op AB's only on post op infection, wound dehiscence, EIF.	2. Ethical approval- Jordan University<br>3. Inclusion- Pt's Oral surgery dept for dental implants from 11.2005-6.2010. >16 years old with missing teeth.<br>4. Exclusion - Radiotherapy head and neck, bacterial endocarditis risk, pen allergy, chronic acute	1. Final sample size was 240 due to high drop out. Due to 14 did not receive allocated intervention, 7 did not show up to post op visit, 9 did not adhere to post op dose.<br>2. Power calculation wanted to get to 80% to detect 1% increase

			Pt: 79<br>Delivery: tablet<br>Type: amoxicillin<br>Onset dose: 2g<br>Onset: preoperatively + postoperatively<br>Continuation dose: 500mg<br>Frequency: 3/day<br>Duration: 5 days					infections close to site, under AB's already, on IV bisphosphonates, pregnancy/lactating mothers.<br>7. Experience- consultant other measurements by OMFS resident.	in dental implant success rate but sample size was 2808 so limited to 270 patients instead so power level reduced. Final size was even lower at 240<br>3. Bias -lack of double blindness<br>8. Funding - Not stated within hospital service.
			Pt: 88<br>Delivery: tablet<br>Type: amoxicillin + clavulanic acid<br>Onset dose: 625 mg<br>Onset: postoperatively<br>Continuation dose: 625 mg<br>Frequency: 3/day<br>Duration: 5 days						
Caiazzo et al., 2011 Italy	RCT II	Total: 100<br>male: 42<br>female: 58<br>implants: 148<br>Age: NR	Pt: 25<br>Delivery: tablet<br>Type: amoxicillin<br>Onset dose: 2g<br>Onset: preoperatively<br>Continuation dose:<br>Frequency:<br>Duration:	1,2,4,8 weeks post op and 3/12	Not defined	A pilot study to determine the effectiveness of different Am regimens in implant surgery.<br>Internal and external oedema, internal and external erythema, pain, heat, and exudate. Implant failure	1. No SS between the 4 groups<br>2. Each groups sample size were too small as stated by authors to detect SS.	2. Inclusion- 2 private practices 9.2006-9.2007<br>3.Exclusion - Systemic disease, long term NSAID use, Medical AB prophylaxis req, Pen allergy, Pregnancy, unwilling to give consent, not attending follow-ups.<br>4. Experience - 2	1. Power was low 15% -confidence 99% 35% confidence 95%<br>2. 5. Bias- Radomisation by computer but not stated if operators were blinded or the patients<br>3. Ethical approval- None stated.

			Pt: 25<br>Delivery: tablet<br>Type: amoxicillin<br>Onset dose: 2g<br>Onset: preoperatively + postoperatively<br>Continuation dose: 1g<br>Frequency: 2/day<br>Duration: 7 days					calibrated experienced surgeons.<br>6. Funding - Not stated but private practice.<br>8.Adverse effects- None noted	
			Pt: 25<br>Delivery: tablet<br>Type: amoxicillin<br>Onset dose: 1g<br>Onset: postoperatively<br>Continuation dose: 1g<br>Frequency: 2/day<br>Duration: 7 days						

Esposito et al., 2010 UK	RCT II	Total: 506<br>male: 236<br>female: 270<br>implants:<br>Age: 48.35 (18-86)	Pt: 252<br>Delivery: tablet<br>Type: amoxicillin<br>Onset dose: 2g<br>Onset: preoperatively<br>Continuation dose: None<br>Frequency: None<br>Duration: None	1,2 weeks, 4 months post op	All types were allowed from flawless to immediate	Effectiveness of prophylactic antibiotics at placement of dental implants :a pragmatic multicentre placebo controlled randomised clinical trial Outcome measures were prosthesis and IF, adverse events and post-operative complications. Patients were seen 1 week, 2 weeks and 4 months post-operatively.	1. No SS differences were observed, although trends clearly favoured the AB group.<br>2. Immediate post-extraction implants were more likely to fail. compared with patients receiving delayed implants (9% versus 2%).	2. Inclusion- Pt's from 4.2008-11.2009 including smokers - non smokers, light smokers and heavy 0,<10 ,>10<br>3.Exclusion -<18, use of AB's in previous 3/12, immunodeficiency, bleeding, allergy to Pen, diabetes, radiotherapy to head neck, implanted biomaterials, bone grafting required, already on antibiotics, on IV bisphosphonates, pregnancy/lactating, enrolled in other study which could affect present trial.<br>4. Experience - private clinics highly experienced clinicians.<br>5. Bias- double blind plus statistician.<br>6. Funding - No sponsorship of any kind<br>7. Ethical approval- Non stated but CONSORT statement<br>8. Adverse effects- None recorded	1. Power - underpowered to detect SS. 2. Corsodyl 0.2% given post op 1min BD 7/7

Anitua et al., 2009 Spain	RCT II	Total: 105<br>male: 35<br>female: 70<br>implants:<br>Age: 47.99 ± 11.98	Pt: 52<br>Delivery: tablet<br>Type: amoxicillin<br>Onset dose: 2g<br>Onset: preoperatively<br>Continuation dose: None<br>Frequency: None<br>Duration: None	3, 10 days 1 month, 3 months post -op	Single implants	A multicentre placebo-controlled RCT of AP for placement of single dental implants.<br>Outcome measures were post-operative infections, adverse events and IF.	1. There were no SS differences for postoperative infection, adverse events, IF<br>2. The use of Am did not either alter or modify the characteristics of the saprophytic flora nor provoke remarkable side effects.<br>3. AP may not be needed in type II or III bone, single implant covered in PGRF	Experience- surgeons and prosthodontists all experienced.<br>Bias- double blind<br>Ethical approval- Basque country ethics committee<br>Adverse effects-None stated	1. Implants had humidification with PGRF prior to installation<br>1. Power - Underpowered should have been 240 ended up with 105<br>2. Use of Chlorhexidine 0.2% as a pre rinse<br>3. Used Dexamethasone post op.<br>Funding- provided by BTI company Spain

Esposito et al., 2008 UK	RCT II	Total: 316<br>male: 142<br>female: 174<br>implants: 696<br>Age:	Pt: 158<br>Delivery: tablet<br>Type: amoxicillin<br>Onset dose: 2g<br>Onset: preoperatively<br>Continuation dose: None<br>Frequency: None<br>Duration: None	1,2,4 weeks and 4 months post op implant tested with 28Ncm torque	Operators allowed to place all types to immediate. Without bone augmentation	Efficacy of AP for dental implants: a multicentre placebo-controlled RCT<br>Outcome measures were<br>1. Prosthesis failure<br>2. IF.<br>3. Post op adverse events<br>4. Post op biological complications	1. No SS differences were observed. However, x4 more patients in the placebo group experienced IF then in the AB group, and this requires further investigation.	1. Inclusion - 9.2006 non smokers, light smokers heavy smokers<br>Exclusion-<18, use of AB's in previous 3/12, immunodeficiency, bleeding, diabetes, radiotherapy to head neck, implanted biomaterials, bone grafting required, already on antibiotics, on IV bisphosphonates, pregnancy/lactating, enrolled in other study which could affect present trial.<br>Experience-12 Italian private clinics experienced clinicians<br>Bias- double blind and statistician was also blinded<br>Funding- No commercial sponsorship<br>Ethical approval- None stated.<br>Adverse effects- 1 in placebo group and 1 in antibiotic group	2. Clindamycin was used as an alternative<br>3. Smokers included.<br>4. Ethical approval- None stated.<br>5. Adverse effects- 1 in placebo group and 1 in antibiotic group<br>6. Under powered author admits the study was underpowered.<br>7. AB's and Placebo donated by Mark Generics Italia.

Abu-Ta'a et al., 2008 Belgium	RCT II	Total: 80<br>male: 43<br>female: 37<br>implants: 247<br>Age: 26-88	Pt: 40<br>Delivery: tablet<br>Type: amoxicillin<br>Onset dose: 1g<br>Onset: preoperatively + postoperatively<br>Continuation dose: 500mg<br>Frequency: 4/day<br>Duration: 2 days	7-10 days 5 months to check for IF	Not stated if with bone grafts	Asepsis during periodontal surgery involving oral implants and the usefulness of peri-operative antibiotics: a prospective, randomised, controlled clinical trial.<br>This RCT compares the usefulness of pre- and post-operative antibiotics while strict asepsis was followed during periodontal surgery.<br>Objectives:Post op infection, implant loss, pain microbiological tests for peri-oral area and nasal samples.	1. No significant differences for post -op infections.<br>2. Does not reduce peri-oral microbial contamination.<br>3. Reduction in post op discomfort with AB's 4. Implant survival AB group =100% v 96% non AB group but cofounding factor of smoking and bruxism	4. Not mentioned type of implant placements.<br>Exclusion-allergy to penicillin, need for endocarditis prophylaxis, any systemic or local immunodeficiency, uncontrolled diabetes mellitus, or previous radiation therapy in the head and neck area. Such patients would systematically receive prophylactic antibiotics and were not considered for randomisation.<br>Bias- Double blind<br>Funding- No external funding just support from University<br>Ethical approval- University Hospital of the Catholic University Leuven.<br>Adverse effects - None stated	1. Power- No power analysis carried out. 2. Experience- Not stated<br>3. Smokers included but not classified into groups low or high smokers<br>4. Very little detail of type of surgical placement - immediate, delayed submerged, flapless etc.

Alsaadi et al., 2007 Belgium	Cohort Study III	Total: 283<br>male: 96<br>female: 187<br>implants: 720<br>Age: 56.2	Pt: 148<br>Delivery: tablet<br>Type: NR<br>Onset dose: NR<br>Onset: NR<br>Continuation dose: NR<br>Frequency: NR<br>Duration: NR	2004 consecutive patients from 1982-2003	Branemark<br>Machine n=6316<br>Tiunite. n=630<br>Not stated just protocol one assumes all types of placements were carried out since not specified.	Impact of local and systemic factors on the incidence of oral implant failures, up to abutment connection	1. Ti-Unite surface v machine no SS 2. 3.6% EIF 1-6 months post placement<br>3. EIF smoking and inc of the number esp > 20<br>4. Periotest readings indicated more failures if lower rigidity<br>5. More EIF with wide platform implants >5mm - but these implants were used as rescue implants and so were more likely placed in soft and resorped bone, there were issues with design and placement<br>6. More EIF with short <10mm implants 7. More EIF in post regions of max and mand v anterior 8. More EIF if implants next to teeth V edentulous<br>9. More EIF in poor quality E grade bone- extreme resorption<br>10. More EIF in grade 4 soft bone<br>11. More EIF in Crohn's, osteoporosis		Power- this is retrospective study<br>Experience- various surgeons with differing abilities<br>Exclusion- None<br>Bias- Retrospective only looking at past data high heterogeneity<br>Funding-Not stated<br>Ethical approval- Not stated<br>Adverse effects- Not stated

Binahmed et al., 2005 Canada	Cohort Study III	Total: 215<br>male: 113<br>female: 104<br>implants: 747<br>Age: 57.15 (18-85)	Pt: 125<br>Delivery: tablet or IV<br>Type: penicillin G or clindamycin<br>Onset dose: 1,000,000 units<br>Onset: preoperatively<br>Continuation dose: NR<br>Frequency: NR<br>Duration: NR<br><br>Pt: 90<br>Delivery: tablet<br>Type: penicillin V or clindamycin<br>Onset dose: 300mg or 150mg<br>Onset: postoperatively<br>Continuation dose: NR<br>Frequency: NR<br>Duration: NR	1, 2 weeks and upto 4 months or prior to uncovery	2 stage protocol, graft not stated, implant type not stated,	Single preoperative dose versus long-term AP regimens in dental implant surgery.<br>prospective study was to compare the efficacy of AP regimens commonly used in dental implant surgery. Preoperative single-dose and long-term prophylactic antibiotic regimens were compared.<br>Post operative infection was looked at	1. Long-term prophylactic antibiotic use in implant surgery was of no advantage or benefit over a single-dose preoperative antibiotic regimen in this patient population.	Inclusion-.18 years old, willing to participate, partially edentulous in either mandible<br>Exclusion-medical condition requiring prophylactic antibiotics, radiation treatment, untreated dental disease, in adequate bone, bone grafting alcoholism, drug abuse and control diabetes and pregnancy<br>Experience- More than 1 surgeon at each site<br>Ethical approval- University of Manitoba and Ohio State University<br>Adverse effects- None stated.	1. Why was Penicillin compared to Amoxicillin which is most common AB 2. IV and oral doses given depending on pt receiving IV sedation<br>3. Clindamycin given to allergic patients either IV or oral<br>4. Power calculation not stated<br>5. Bias- Lack of randomisation<br>6. Funding-Not stated

Morris et al., 2004 USA	Cohort Study III	Total: 0<br>male: 0<br>female: 0<br>implants: 1500<br>Age: NR	Pt: NR<br>Delivery: tablet<br>Type: Mix<br>Onset dose: NR<br>Onset: NR<br>Continuation dose: NR<br>Frequency: NR<br>Duration: NR	3-5 year period	Ankylos implants oinly	The influence of AB use on the survival of a new implant design	1. No SS in IFR between with without AB coverage 2. Pre-op AB's survival 95.2%<br>3. Adequate AB's 95.7% -AHA-90<br>4. Inadequate AB's 98.1% -AHA-90 5. Adequate 95.9% - AHA-97<br>6. Inadequate 97.2% -AHA-97<br>7. Adequate 96.5% - Petersons recommendations<br>6. Inadequate 96.3% - Petersons recommendations 8. Post opAB's 97%	Inclusion-None stated<br>Exclusion- None stated<br>Experience- Various evaluators<br>Ethical approval- None stated<br>Adverse effects- Not stated	Power- None stated however 1500 implants studied not sure if multiple in 1 pt.<br>2. Bias- Not randomised AB's depending on clinicians preference no blinding of the surgeon or patient<br>Funding- Ankylos Implant clinical Research group- Friadent GmbH, Mannheim, Germany (formerly Degussa-AG, Hanau, Germany)<br>Ethical approval- None stated<br>Adverse effects- Not stated

Laskin et al., 2000. USA	Cohort Study III	Total: 702 male: female: implants: 3030 Age:	Pt: 387<br>Delivery: tablet<br>Type: Mix<br>Onset dose: NR<br>Onset: preoperatively<br>Continuation dose: None<br>Frequency: None<br>Duration: None	Upto 36 months (3 years)	Treatment strata:<br>1) lower completely edentulous;<br>2) upper completely edentulous; 3) lower posterior partially edentulous; 4) upper posterior partially edentulous; and 5) upper anterior single tooth,<br>Hydroxyapatite coated implants of various designs and non coated of various designs	The influence of Preoperative AB's on success of endosseous implants at 36 months.<br>The purpose of this study was to assess the influence of preop AB's on long-term clinical survival of endosseous dental implants of different designs and surfaces.<br>Outcomes Implant survival rates looking at surgeon experience, chlorhexidine, implant coating, bone density, incision type, mobility at placement, implant design, type of AB regimen	1. IFR was 4.6% v 10% with and without pre-op AB's 2. AHA recommended 95.3%<br>3. Inadequate or no pre-op AB's = 92% 4. Petersons criteria 95.7% v 91.1% low dose or no pre-op AB's.	Inclusion- None stated<br>Exclusion- None stated<br>Experience- Compared data with <50 and >50 implants previous experience<br>Funding-Dental Implant Clinical Research Group (DICRG<br>Ethical approval- None stated<br>Adverse effects- None stated	1. No standardisation on antibiotic types pre- or post<br>Power- Not calculated but n=702<br>Bias- No blinding, no randomisation as AB's were left to discretion of the clinician.<br>8. Majority of patients received post op AB's at 96%

LEGEND: AB – Antibiotic.
